# Long-term Effectiveness of Trabectome (Ab-interno Trabeculectomy) Surgery

**DOI:** 10.5005/jp-journals-10028-1256

**Published:** 2018

**Authors:** Rick E Bendel, Michael T Patterson

**Affiliations:** 1,2 Department of Ophthalmology, Mayo Clinic Foundation of Florida, Jacksonville, Florida, USA

**Keywords:** Intraocular pressure, Minimally invasive glaucoma surgery, Trabectome, Trabeculectomy, Retrospective chart review

## Abstract

**Aim:**

To evaluate the long-term safety and efficacy of ab-interno trabeculectomy with trabectome for the treatment of glaucoma.

**Materials and methods:**

Data collected for 339 eyes which included demographics, intraocular pressure (IOP) measurements using Goldmann applanation tonometry, best-corrected visual acuity (BCVA), visual field results, optic nerve status, gonioscopic findings, prior glaucoma procedures, number of glaucoma medications and pain level. The main data points of interest were preoperative IOP *vs.* postoperative IOP and BCVA, medication use, pain status, and complications.

**Results:**

Of the 339 eyes that underwent trabectome, we found a statistically significant reduction in IOP (*p* < 0.01) at final follow-up (average = 18.35 months) of nearly 23%, with a complication rate of 5.86%. Furthermore, this reduction was maintained up to 8 years post procedure. LogMAR visual acuity was significantly improved in 69% of eyes at the final visit (*p* < 0.05), while only 1.77% of cases saw a significant reduction. Based on these findings, we determined a success rate of around 80% to 100 months following trabectome.

**Conclusion:**

Trabectome is a safe and effective long term for most forms and severities of glaucoma.

**How to cite this article:**

Bendel RE, Patterson MT. Long-term Effectiveness of Trabectome (Ab-interno Trabeculectomy) Surgery. J Curr Glaucoma Pract 2018;12(3):119-124.

## INTRODUCTION

Glaucoma is the second leading cause of irreversible blindness worldwide.^1,2^ Current proven surgical techniques to treat glaucoma only slow the progression of optic nerve damage by reducing intraocular pressure, rather than reversing the damage. These surgeries typically require either an implant or filtration style procedure. However, implant methods are invasive and classic filtration methods, such as trabeculectomy, result in a high complication rate.^3^

Ab-interno Trabeculectomy with Trabectome (Neomedix Corp) remains a promising, minimally invasive glaucoma procedure that decreases IOP by carefully ablating and removing some of the Trabecular meshwork and exposing collector channels, which facilitates aqueous humor outflow.^4,5^ The Trabectome is a surgical tool that was approved for use by the US Food and Drug Administration in 2004,^6^ and gained popularity for the treatment of various forms of glaucoma.^7^ The Trabectome procedure presents a viable alternative to traditional trabeculectomy because of the absence of an external filtering bleb. Bleb failure and scarring remains one of the most prevalent complications associated with filtration surgeries and bleb maintenance requires lifelong follow-up postoperatively.

Many studies highlight the short-term IOP lowering ability, and low complication rate of trabectome,^8–11^ however, long-term data on its effectiveness and patient satisfaction remains scarce. In this retrospective chart study, we evaluate the long-term efficacy and safety of trabectome in patients with various types of glaucoma.

## MATERIALS AND METHODS

A case series of subjects who had undergone ab-interno trabeculectomy with the trabectome from March of 2008 to June of 2016 was analyzed with the approval of the institutional review board and by the Declaration of Helsinki and the Health Insurance Portability and Accountability Act. All patients were previously diagnosed with glaucoma, and a single surgeon performed all procedures at the Mayo Clinic of Florida in Jacksonville.

Preoperative data was collected for 339 eyes which included demographics, IOP measurements using Goldmann Applanation Tonometry, BCVA, visual field results, optic nerve status, gonioscopic findings, prior glaucoma procedures, and many glaucoma medications. Postoperative data was collected for the following parameters: IOP, BVCA, complication status following an ocular exam, glaucoma medications and pain level using a verbal analog scale where patients were asked to gauge their pain as either no pain, mild pain, moderate pain or severe pain. Demographic data are summarized in Table 1 .

**Table 1:** Patient demographics and ocular status of all eyes that underwent trabectome

*Patient demographics*	*Percentage (%)*
Number of eyes	339
*Age*	
Mean age	75.5
Range	36–93
Sex	
Male	109
Female	92
*Race*	
Caucasian	279
African American	33
Asian	8
Other	19
*Glaucoma type*	
POAG	206
Pseudoexfoliation	20
Pigmentary	9
Narrow-angle (NAG)	52
Trauma	2
Unspecified	43
Low tension	4
*Visual acuity*	
Average preoperative	
*Visual field*	
Mild	20/40.8
Moderate	201
Severe	98
*Cup to disk*	
<0.5	40
0.5–0.69	31
0.7–0.79	115
0.8–0.89	75
>0.9	72
*Prior surgery*	
LTP	46
Trabeculectomy	107
PI	10
AVG	28
*Angle: Shaffer grade*	
0	10
1	15
2	79
3	45
4	60
	140

The main data points of interest were preoperative IOP *vs.* postoperative IOP, medication use, pain status, and complications. The average follow-up time was 18 months (range 3–100 months). Patients were diagnosed with the following types of glaucoma: pseudoexfoliation, primary open angle, narrow-angle, trauma, low tension, pigmentary, and other secondary forms of glaucoma (Table 1). The inclusion criteria were: the presence of uncontrolled glaucoma on maximum tolerated glaucoma medications. For patients who had a preoperative IOP > 15, success was defined as a postoperative IOP between 5 to 20 mm Hg, no increase in the number of glaucoma medications and no reduction of visual acuity. For patients who had a preoperative IOP < 15, success was defined as an increase in visual acuity, no increase in IOP and no increase the number of glaucoma medications. Failure was a result of not meeting the success parameters or occurrence of prolonged pain and/or complications. Statistical analysis of the difference in IOP and number of glaucoma medications before and after surgery were carried out using a paired t-test where a *p* value of < 0.05 was considered statistically significant. A Kaplan–Meier analysis was employed to determine survival rates using our success definition to compensate for the varying follow up times. All statistical analyses were performed using GraphPad Prism.

In the operating room, brimonidine, antibiotic, and topical anesthetic drops were instilled in the operative eye. Then, the eye was draped using standard sterile technique. A temporal corneal incision was made, and lidocaine was injected into the anterior chamber (AC). Trabectome ablation of approximately 120 to 160 degrees of the trabecular meshwork was performed under gonioscopy using the NeoMedix Trabectome Goniolens. The patient's head was tilted 30 to 40 degrees away from the surgeon, and the microscope was also tilted 30 to 40 degrees toward the surgeon during each procedure. When combined with cataract removal and lens implant (IOL), the trabectome was always performed first. Any blood reflux was cleared from the AC. Carbachol was then injected into the AC, and the incisions were sealed with BSS. Dexamethasone was then injected subconjunctivally, and antibiotic drops were reapplied. When synechial closure was present, this area of the trabecular meshwork was avoided. The patient was positioned to the greatest extent possible to avoid synechial areas. During preoperative evaluation, if more than 1 clock hour of synechial closure was present, trabectome was not performed.

## RESULTS

**Graphs 1A to F:**
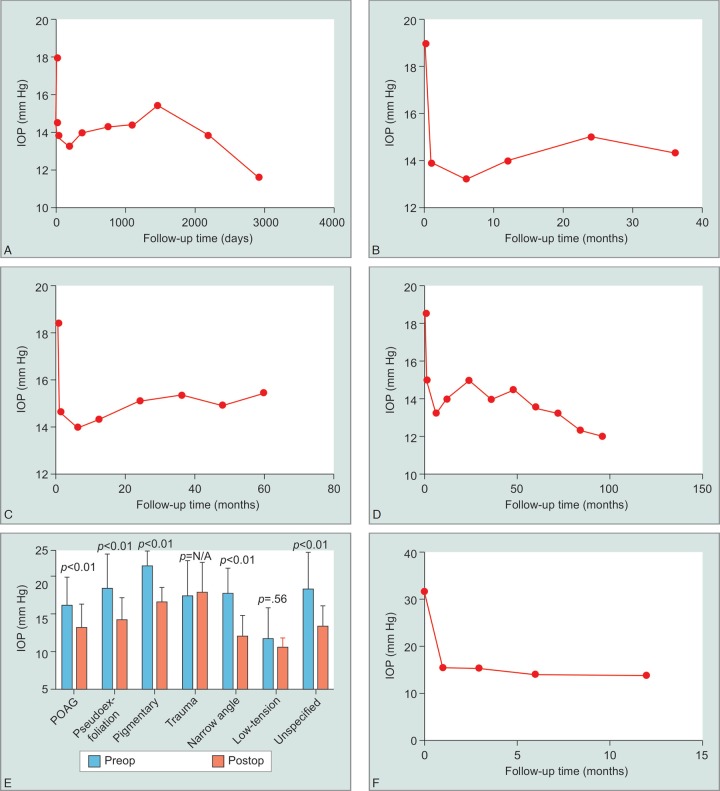
Intraocular pressure data. (A) Intraocular pressures of all eyes that underwent trabectome procedure at various follow-up times; (B) Intraocular pressures at various time points for patients who had 3 years of follow-up data; (C) Intraocular pressures at various time points for patients who had 5 years of follow-up data; (D) Intraocular pressures at various time points for patients who had 8 years of follow-up data; (E) Intraocular pressures before trabectome and at last follow-up visit after trabectome stratified by type of glaucoma; (F) Intraocular pressures at various time points after trabectome for the eyes that had preoperative IOPs greater than 30 mm Hg

A total of 339 eyes were enrolled in this study that had been previously diagnosed with various forms of glaucoma. Mean IOP before undergoing trabectome was 18.01 ± 1.2 mm Hg and was significantly reduced to 13.89 ± 1.5 mmHg at the final follow-up visit (*p* <0.01). The average reduction in IOP at the final follow-up was 19.62%. Graph 1A compares preoperative IOP to posoperative measurements at various follow-up times. To assess the ability of eyes to maintain a long term stable IOP after undergoing trabectome, we looked specifically at patients who had follow-up data of 3, 5 and 8 years, and found each cohort had a statically significant reduction at final follow-up (*p* <0.01) (Graphs 1B to D). Graph 1E shows the average reduction of IOP stratified by type of glaucoma. All groups saw a statically significant reduction of IOP at the final follow-up visit, except the low-tension and trauma patients, which had limited follow-up data. Interestingly, we saw the largest decrease in IOP in patients who had been diagnosed with narrow-angle glaucoma. Furthermore, we also explored how effective this procedure was at lowing IOP in refractory cases (preoperative IOP >30 mm Hg) and saw a mean reduction of 15.53 mm Hg (42.34%), which was statistically significant (*p* <0.01) (Graph 1F).

**Graph 2:**
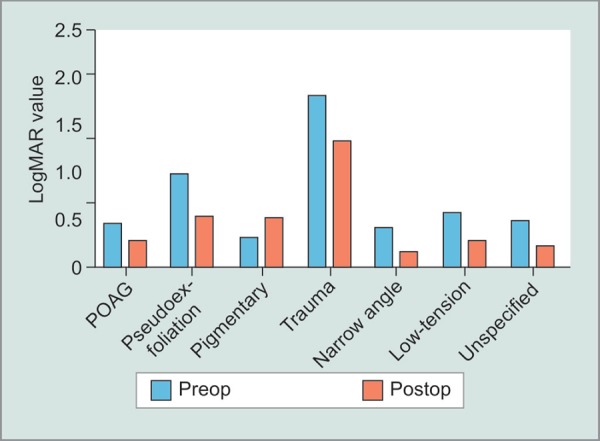
Average LogMAR visual acuity prior to trabectome and at final follow-up visit after trabectome stratified by type of glaucoma

**Graph 3:**
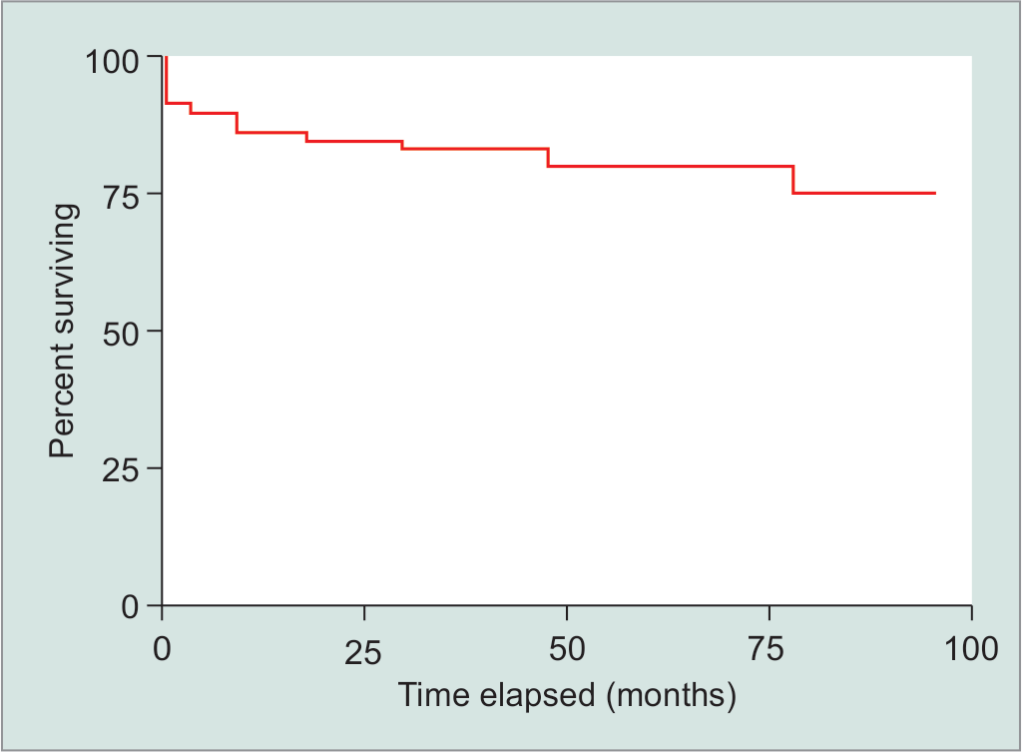
Kaplan–Meier survival curve of all patients who underwent trabectome

**Graphs 4A and B:**
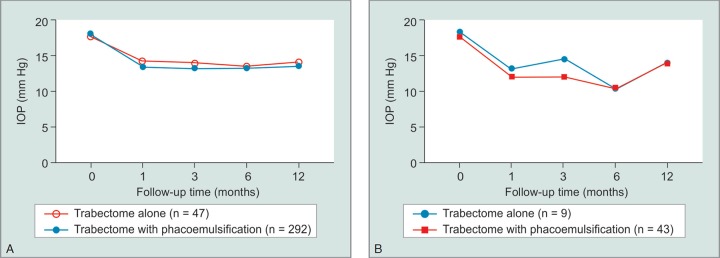
(A) Intraocular pressures at various follow-up times, comparing eyes that underwent phacoemulsification in conjunction with trabectome vs eyes that received trabectome alone; (B) Intraocular pressures at various follow-up times, comparing eyes that underwent phacoemulsification in conjunction with trabectome vs eyes that received trabectome alone specifically within the angle closure group

To assess visual outcomes, best corrected visual acuity was expressed on a log MAR scale, and preoperative and postoperative results were compared by type of glaucoma (Graph 2). We found that 68.93% of eyes saw an improvement of BCVA, 29.30% saw no significant change and 1.77% saw a decrease in at the final follow-up visit. The number of preoperative and postoperative glaucoma medications were also compared, and we saw an average decrease from 1.73 medications to 1.13 at the final visit. A paired t-test was used to compare the statistical significance of this finding and a *p* value of <0.05 was determined (*p* = 0.03). Minor complications due to the procedure occurred in 20 (5.96%) of the cases while 0 cases presented significant complications (Table 2). Kaplan–Meier survival analysis was used to determine success based on follow-up time (Graph 3) and showed stable rates up to 100 months postoperatively around about 80%. Of interest, there was no statistical difference in the IOP outcomes after trabectome with or without IOL, including eyes with narrow angles (Graph 4) (*p* = 0.64).

Postoperative pain was one important variable assessed in this study and only 3 (0.88%) of patients reported prolonged issues with pain (Table 2). To determine the success rate of trabectome, patients were split into two groups based on their average preoperative IOP. Eyes that had a preoperative IOP > 15 mm Hg saw success in 85.95% of cases two years after trabectome while eyes that had a preoperative IOP < 15 achieved success in 83.99%, giving an overall success rate of 84.96% two years postop. Kaplan–Meier survival analysis was used to determine success based on follow-up time (Graph 3) and showed stable rates up to 100 months postoperatively of about 80%.

**Table 2:** Complications and additional procedures required after Trabectome

*Complications*	
Uncontrolled IOP (35 mm Hg+)	7 (2.06%)
CME	9 (2.65%)
Hyphemia	16 (4.72%)
Conjunctival hemorrhage	5 (1.47%)
Prolonged pain	3 (0.88%)
Additional procedures needed	
TSCPC	2 (0.59%)
Valve placement	3 (0.088%)
Laser trabeculoplasty	1(0.29%)

## DISCUSSION

In this study, we evaluated the long-term outcomes of ab-interno trabeculectomy with trabectome to gain insight on how effective this surgery is in stopping or slowing the progression of glaucoma for many years after the operation.

Our cohort of patients, on average, had a lower preoperative IOP than similar studies,^6,9,12^ suggesting that other factors than elevated IOP, such as poor visual acuity and dependence on glaucoma drops, merited surgical intervention. Patients saw an average IOP decrease of 19.58% at their final visit, which further affirmed the IOP lowering the ability of trabectome highlighted in other studies.^9,12–14^ We also saw a similar average decrease in glaucoma medication at the final follow-up after surgery to many previous studies.^9^ More importantly, we found that in patients who had long term follow-up data, a stable IOP was maintained up to eight years. Of the various types of glaucoma diagnosed, we saw the largest average IOP decrease in eyes with narrow-angle glaucoma. Many surgeons hesitate to perform this type of drainage surgery in narrow-angle patients due to the perceived increased risk,^15^ however Bussel et al. recently showed that there was no correlation of increased risk or decreased effectiveness based on Shaffer grade and our study further affirms this.^5^ In comparing our findings to similar series, we found few studies that reported outcomes past eighteen months post-operatively.^16,17^ Ahuja et al. published a two-year case study from a cohort of patients who underwent trabectome and found that only 22% maintained a postoperative IOP ≤ 18 mm Hg after two years and suggested that trabectome is only appropriate for patients requiring a target IOP of 21 mm Hg or above,^17^ while we found that about 80% of our patients saw an IOP of ≤ 18 mm Hg at two years. Our lower average preoperative IOP could account for the difference in outcomes; however, we believe that our large sample size strongly suggests that the trabectome procedure is successful in maintaining long-term, stable IOPs. Hashemian et al. recently reported in a 30 patients cohort with similar average preoperative IOP to our study, that trabectome was effective at lowering and maintaining IOP 12 months postoperatively.^18^ One limitation of this study was the small sample size; however, we believe that our data further affirms the findings of their study and highlights our conclusion that trabectome is effective even in cases where elevated IOP may not be the main indicator of a need for surgical intervention.

While many studies focus on the IOP lowering ability of IOP with trabectome,^5,9,10^ there is a lack of data on both visual outcomes and overall patient satisfaction. We report that in almost 70% of cases there was an improvement of visual acuity, while only 1.77% of eyes saw a substantial decrease in vision. Most cases that saw significant vision loss were rather complex and had undergone multiple prior failed glaucoma procedures. Of the few previous comparable studies done assessing visual outcomes after trabectome, Lee et al. reported in a cohort of Chinese patients with open-angle glaucoma that visual acuity was stable six months postoperatively,^19^ further confirming our claim.

Regarding long-term patient satisfaction after trabectome, we assessed complication rates, additional surgeries needed and reported prolonged pain. Only 4.17% required further surgical intervention, which is much lower than previously reported data.^8,14,20^ Of the cases that required additional procedures, over half were due to an inability to maintain a stable IOP. With regards to complication and prolonged reported pain rates, we found that 10.9% of cases saw minor complications and only 0.88% of patients reported mild to severe pain at two consecutive visits after trabectome. Taking all this data into consideration suggests that long term patient satisfaction is high.

In comparing our success rates to previous studies on trabectome, we found a one-year success rate of around 85%, which was similar to published data.^9,21–24^ However, we found much higher success rates compared to the few studies that assessed outcomes over one year.^13–14^ When defining success, we found it useful to divide our cohort into two groups based on their preoperative IOP because it allowed for us to assess outcomes where an elevated IOP was not the main indicator of a need for surgical intervention. Furthermore, when we assessed the IOP lowering ability of trabectome in patients who had a preoperative IOP greater than 30, we found that in most these cases, multiple previous surgeries were attempted and failed thus giving us insight into trabectome's effect on refractory cases. We found that these patients had a significant decrease in IOP and maintained this up to a year postop. Roy et al. recently reported in a retrospective chart review of 498 cases, that IOP reduction is correlated to glaucoma severity.^25^ We believe that our study is in line with this claim; additionally, we also suggest that trabectome is also effective in less advanced cases.

## CONCLUSION

Our study suggests that ab-interno trabeculectomy with trabectome is a safe and effective tool for maintaining a stable IOP and visual field up to 8 years. We conclude that trabectome should be considered in a wide variety and severity of glaucoma cases.

## CLINICAL SIGNIFICANCE

While trabectome has been proven to be an effective procedure for lowing IOP in the short term, long term data has yet to be established. Furthermore, minimal data has been reported about the visual outcomes of patients after trabectome. This study provides one of the longest duration of follow up after trabectome surgery published with minimal loss in success out to eight years and great stability of glaucoma control. Our work makes it very clear that regardless of the type or severity of glaucoma, trabectome has an essential place in the surgical options for most glaucoma patients.
